# Age and cohort trends in disability among Chinese older adults

**DOI:** 10.3389/fpubh.2023.998948

**Published:** 2023-03-08

**Authors:** Chaoping Pan, Na Cao, Mohammedhamid Osman Kelifa, Shuren Luo

**Affiliations:** ^1^School of Public Health, Wuhan University, Wuhan, Hubei, China; ^2^The Second Affiliated Hospital of Guangxi Medical University, Nanning, Guangxi, China

**Keywords:** older adults, A-P-C model, disability, disablement process model, China

## Abstract

**Objective:**

This study aimed to examine age and cohort trends in disability among Chinese older adults and explore the disablement process factors that may explain the cohort trends in disability.

**Methods:**

This study used data from five waves of the Chinese Longitudinal Healthy Longevity Survey (CLHLS). A hierarchical logistic growth model was used to analyze the A–P–C effects and the contributors of cohort trends.

**Results:**

ADL, IADL, and FL among Chinese older adults showed increasing age and cohort trends. FL was more likely to result in IADL disability than ADL disability. Among the disablement process factors, gender, residence, education, health behavior, disease, and family income contributed to most of the cohort trends in disability.

**Conclusions:**

As older adults face increasing disability trends, it is necessary to distinguish age and cohort trends and develop more effective interventions according to relative contributors to prevent disability among them.

## Introduction

In recent years, the development of economy and medical care systems have largely expanded life expectancy. However, a longer life expectancy also made older adults suffer more from disability. Preventing disability can reduce huge healthcare expenditure, hospitalization, and mortality rates (1). China has the largest population of older adults in the world and the country is aging much faster than other countries (2). This may cause a large number of older adults to live with disabilities. In 2020, more than 40 million Chinese older adults were disabled, and it was estimated that disabled older adults in China will reach 65 million by 2030 (3). Therefore, more research into disability patterns and related determinants among older persons is required to develop more effective interventions to alleviate the financial burden of healthcare costs on families and society.

In the field of demography, time can be captured by three temporal dimensions: age, period, and cohort (A–P–C) (4). Each aspect of A–P–C makes a unique contribution to population health. Age (A) is an indicator of biological processes that cause internal physiological changes and eventually lead to morbidity, disability, and/or death. Period (B) describes morbidity, disability, or/and death at a given time and reflects economic, sociocultural, and technological aspects. Cohort (C) captures the health status of successive generations who are born in a social system during a similar period of time and experience similar social experiences in their lifetime (4–6). Failure to distinguish A–P–C trends may lead to a non-negligible bias and provide an incomplete analysis of population health trends (7).

Though many studies explored the trends in disability, only a few studies explored the A-P-C trends in disability among older adults (4, 6, 8, 9). The results consistently displayed an increasing age trend. However, the period and cohort trends were more equivocal. Lin et al. found decreasing period and cohort trends for both Blacks and whites among the oldest-old in America by studying a cross-classified random effect model (4). Lin et al. also found a decreasing period trend and an increasing cohort trend among people aged 70 years and over in America by performing a fixed-effects approach (6). A study conducted in Hong Kong showed an increasing period trend of ADL disability and no cohort effect among Chinese older adults (9). Zhang indicated an increasing period trend and a declining cohort trend among the oldest-old in China using the intrinsic estimator method (8). However, this study was focused on the oldest-old, which was too old to represent the older population in China. The differences in cultural context, age, indicators of disability, or methods used in the analysis may account for the inconsistent results of previous studies. So, further studies are needed to explore the A–P–C trends in disability among Chinese older adults.

The disablement process model proposed by Verbrugge and Jette in 1994 may help us to understand the process involved in disability (10). According to the disablement process model, disability, defined as difficulties in the performance of socially defined roles and tasks, is commonly measured by the Activity of Daily Living (ADL) and the Instrumental Activities of Daily Living (IADL) and reflects the Functional Limitation (FL) in real-life contexts. FL refers to a disability, independent of the situational requirement. Recently, more researchers recognized that FL may improve the evaluation of disability (11, 12).

The disablement process model is widely used to explore the influencing factors of disability among older adults (13, 14). According to the disablement process model, risk factors, accommodating factors, and disease could affect the disability process. For instance, risk factors contained demographic factors and health behaviors. Demographic factors included gender, education, and so on (13, 14). Health behaviors contained smoking, exercising, and so on (15). Accommodating factors included social supports [e.g., spouse, living arrangement, household income, and social participation (SP)] and so on (16, 17). Disease included chronic disease and psychological resilience (18). However, few studies included these variables to explain the cohort and disability trends in disability, especially using multiple indicators of disability.

The current study aimed to examine age and cohort trends in disability among Chinese older adults and explore the disablement process factors that may explain the cohort trends in disability by analyzing survey data from five waves of the Chinese Longitudinal Healthy Longevity Study (CLHLS).

## Methods

### Study design and participants

This study draws on data from five waves (2005, 2008, 2011, 2014, and 2018) of CLHLS. To ensure sample representativeness, the CLHLS randomly selected 23 out of 31 provinces in China, which covered 85% of the total population in China. In the survey, individuals aged 65 years and over were studied, and the baseline number of participants in 2005 was 15,638. Survey details were provided in previous studies (16, 19). The datasets presented in this study are openly available in CLHLS at https://opendata.pku.edu.cn/dataset.xhtml?persistentId=doi:10.18170/DVN/WBO7LK. As there were only a few respondents older than 106 years, this study only focused on individuals 65–105 years old. After excluding those individuals older than 105 years, the numbers of respondents in each wave were as follows: wave 2005, *n* = 15,613; wave 2008, *n* = 16,563; wave 2011, *n* = 9,679; wave 2014, *n* = 7,107; and wave 2018, *n* = 15,771.

In the analysis of longitudinal data, participant attrition is an issue of particular concern. In this study, intermittent missing data were assumed as missing at random (MAR). The multiple imputation (MI) method was efficient to handle MAR missing values; therefore, this study applied the MI method to deal with the missing values of independent variables (20). We also included alive, dead, or lost to follow-up as a categorical variable into the growth A–P–C model to control for any bias due to these statuses.

### Dependent variables

Activity of Daily Living (ADL) was measured at each wave using six items: dressing, bathing, indoor transferring, toileting, continence, and feeding. Participants were asked if they needed assistance with each of the six activities. Individuals were defined as having ADL disability if they needed assistance in performing at least one of the six daily activities.

Instrumental Activities of Daily Living (IADL) was composed of eight items: shopping, visiting neighbors, washing clothes, making food, walking 1 km, crouching and standing (repeated three times), carrying 5 kg weight, and taking public transport. Respondents were categorized as having IADL disability if they needed help in performing at least one of the eight items.

Functional Limitation (FL) was formed by five items: standing up from sitting in a chair, holding a hand behind the neck, holding a hand behind the lower-back, being able to pick up a book from the floor, and holding up arms. Older adults unable to perform at least one of the five items were treated as having FL and were coded as 1 and those who were able to perform all the items were coded as 0.

### Independent variables

#### Time variables

Age was taken as a continuous variable and included in the model after mean centralization to facilitate the interpretation of the intercept. The birth cohorts were established by subtracting individual ages from the survey years and were treated as categorical variables. The values were grouped into groups of 5 years (1906–1945). Those who were born in or before 1905 were or in and after 1946 were combined into one group to gather enough group members.

#### Control variables

##### Disease

Disease included chronic disease and psychological resilience. Chronic disease was a count of self-reported health conditions (ranging from 0 to 12), including diabetes, hypertension, cancer, arthritis, respiratory diseases (bronchitis, emphysema, pneumonia, and asthma), tuberculosis, stroke, cataract, duodenal ulcer, glaucoma, bedsore, and Parkinson's disease.

Psychological resilience was measured by three items, including “look on the bright side of things,” “keep my belongings neat and clean,” and “be happy when younger”. Each question was assessed on four grades: always (0), often (1), sometimes (2), seldom (4), and rarely or never (5). The score for psychological resilience ranged from 0 to 12, with a higher score representing worse psychological resilience (21).

##### Risk factors and accommodating factors

Risk factors included demographic factors (gender and education) and health behaviors (smoking and drinking). Gender was categorized as men (coded as 0) and women (coded as 1). Education was measured by years of education. We classified smoking as having a smoking habit (coded as 1) or not (coded as 0) and classified exercise as having exercise habits (coded as 1) or not (coded as 0).

Accommodating factors included social supports and were measured by co-residence, marital status, family income, and SP. Co-residence was categorized as living alone (coded as 0) and not living alone (coded as 1). Marital status was defined as having a spouse (coded as 1) and not having a spouse (coded as 0). Family income was divided into seven levels: RMB¥ 0~9,999, RMB¥ 10,000~19,999, RMB¥ 20,000~29,999, RMB¥ 30,000~39,999, RMB¥ 40,000~49,999, and RMB¥ 50,000 and above (coded as 5), respectively, coded 0–5. SP was assessed by asking participants the following questions: “Do you play cards/mah-jongg at present?”; “Do you take part in some social activities at present?”; and “Do you travel beyond home county?”. The total score was 0–3, with a higher score representing a better SP.

### Data analysis

Many methods can analyze the A–P–C effects, such as intrinsic estimation, generalized constrained estimation, the cross-classified random effect model, and the hierarchical logistic model (4, 7, 8). In this study, A–P–C effects were analyzed using a hierarchical logistic growth model, which can be employed in the analysis of longitudinal data and does not require specific constraints. The hierarchical logistic growth model classified time-varying variables (e.g., age) into level 1 and time-invariant variables (e.g., gender) into level 2. We conducted a series of hierarchical logistic models to examine age and cohort trends and explored related factors, in which age, residence, co-residence, marital status, smoking, exercising, chronic disease, and psychological resilience were all included in the level 1 part of the model as they are time-varying variables. Cohort, gender, education, and attrition (alive, dead, or lost to follow-up) were included in the level 2 part of the model as they are time-invariant variables. The square term of age was used to estimate the non-linear effects of age. Cohort was included as a categorical variable. Consistent with most previous studies, period was not included in the model due to the collinearity of age, period, and cohort effects (7). We used SAS 9.4 and “glimmix” procedure to perform the analysis. The model is shown as follows.

Level 1:


$$\begin{array}{l} \ln\left(\frac{P_{ij}}{1-P_{ij}}\right)=\beta_{0j}+\beta_{1}age_{i}+\beta_{2}{age_{i}}^{2}\;+\overset{K}{\underset{k=3}{\sum }}\beta_{k}covariate_{ki} \end{array}$$

Level 2:


$$\begin{array}{l} \beta_{0j}=\Pi_{0}+\gamma_{01}cohort_{1j}+\overset{Q}{\underset{q=2}{\sum }}\gamma_{0q}covariate_{qj}+c_{0j} \end{array}$$

To explore the contribution of each element to the disablement process, we added the variables into the model separately. We compared the OR values before and after including each covariate variable separately in the model and then estimated the contribution of each variable (7, 22). The formula is shown as follows.


$$\begin{array}{l} \text{Contributions}=\left(OR_{before}-OR_{after}\right)/\left(OR_{before}-1\right) \end{array}$$

## Results

### Descriptive characteristics

The sample descriptive characteristics based on cohorts are shown in Table 1. The results showed that older adults in more recent cohorts suffered less from ADL, IADL, and FL disabilities. Individuals were younger in more recent cohorts. Women had a higher proportion than men in earlier cohorts, which was narrowed in more recent cohorts. The proportion of urban older adults increased from 44% in the 1905 and earlier cohort to 52% in the 1946 and subsequent years cohort. In more recent cohorts, older adults had better psychological resilience, higher income, higher SP, and better education. They smoked and exercised more as well. Greater proportions of respondents had a spouse in more recent cohorts.

**Table 1:** The sample descriptive characteristics based on cohorts.

**Variables**	**1905 and before**	**1906–2010**	**1911–1915**	**1916–1920**	**1921–1925**	**1926–1930**	**1931–1935**	**1936–1940**	**1941–1945**	**1946 and after**
ADL	0.56 (0.50)	0.50 (0.50)	0.38 (0.48)	0.35 (0.48)	0.24 (0.43)	0.17 (0.37)	0.11 (0.31)	0.07 (0.25)	0.05 (0.21)	0.04 (0.19)
IADL	0.98 (0.15)	0.95 (0.95)	0.91 (0.28)	0.86 (0.35)	0.77 (0.42)	0.66 (0.47)	0.50 (0.50)	0.36 (0.48)	0.29 (0.45)	0.21 (0.41)
FL	0.81 (0.40)	0.75 (0.75)	0.67 (0.47)	0.59 (0.49)	0.48 (0.50)	0.39 (0.49)	0.28 (0.45)	0.20 (0.40)	0.16 (0.37)	0.14 (0.35)
Age	100.45 (4.56)	99.51 (99.51)	95.44 (4.13)	92.52 (5.18)	88.00 (4.79)	83.49 (5.05)	78.34 (5.10)	73.57 (5.14)	71.55 (3.80)	68.46 (2.31)
Gender	0.81 (0.39)	0.73 (0.73)	0.62 (0.48)	0.58 (0.49)	0.52 (0.50)	0.50 (0.50)	0.50 (0.50)	0.49 (0.50)	0.47 (0.50)	0.47 (0.50)
Education	0.16 (0.36)	0.19 (0.19)	0.28 (0.45)	0.32 (0.47)	0.37 (0.48)	0.44 (0.50)	0.50 (0.50)	0.61 (0.49)	0.72 (0.45)	0.78 (0.41)
Residence	0.44 (0.50)	0.40 (0.49)	0.44 (0.50)	0.49 (0.50)	0.44 (0.50)	0.46 (0.50)	0.48 (0.50)	0.49 (0.50)	0.48 (0.50)	0.52 (0.50)
Smoke	0.08 (0.27)	0.09 (0.09)	0.12 (0.33)	0.15 (0.35)	0.17 (0.37)	0.19 (0.39)	0.21 (0.41)	0.24 (0.42)	0.24 (0.43)	0.22 (0.42)
Exercise	0.16 (0.37)	0.18 (0.18)	0.21 (0.41)	0.24 (0.43)	0.27 (0.44)	0.33 (0.47)	0.39 (0.49)	0.41 (0.49)	0.40 (0.49)	0.41 (0.49)
Chronic disease	0.70 (0.90)	0.71 (0.71)	0.73 (0.88)	0.80 (0.92)	0.90 (0.98)	0.99 (1.03)	1.01 (1.01)	1.03 (1.03)	1.04 (1.02)	1.06 (1.00)
Psychological resilience	4.44 (1.75)	4.48 (4.48)	4.43 (1.79)	4.33 (1.81)	4.34 (1.84)	4.22 (1.89)	4.06 (1.91)	3.99 (1.92)	3.90 (1.86)	3.89 (1.94)
Family income	0.73 (1.24)	1.28 (1.28)	1.27 (1.61)	1.56 (1.76)	1.59 (1.78)	1.62 (1.81)	1.56 (1.83)	1.59 (1.81)	2.13 (1.88)	2.58 (1.97)
SP	0.10 (0.35)	0.13 (0.13)	0.18 (0.46)	0.23 (0.53)	0.30 (0.59)	0.39 (0.67)	0.49 (0.73)	0.59 (0.79)	0.68 (0.83)	0.79 (0.89)
Living alone	0.91 (0.28)	0.89 (0.89)	0.84 (0.36)	0.84 (0.37)	0.81 (0.39)	0.80 (0.40)	0.81 (0.39)	0.85 (0.36)	0.86 (0.34)	0.90 (0.30)
Having a spouse	0.03 (0.18)	0.06 (0.06)	0.11 (0.31)	0.16 (0.37)	0.27 (0.45)	0.39 (0.49)	0.51 (0.50)	0.63 (0.48)	0.70 (0.46)	0.78 (0.42)

Standard errors are given in parentheses. Continuous variables are shown as mean (standard deviation). As all classification variables are dichotomous, categorical variables are also presented as mean (standard deviation).

**Figure 1:**
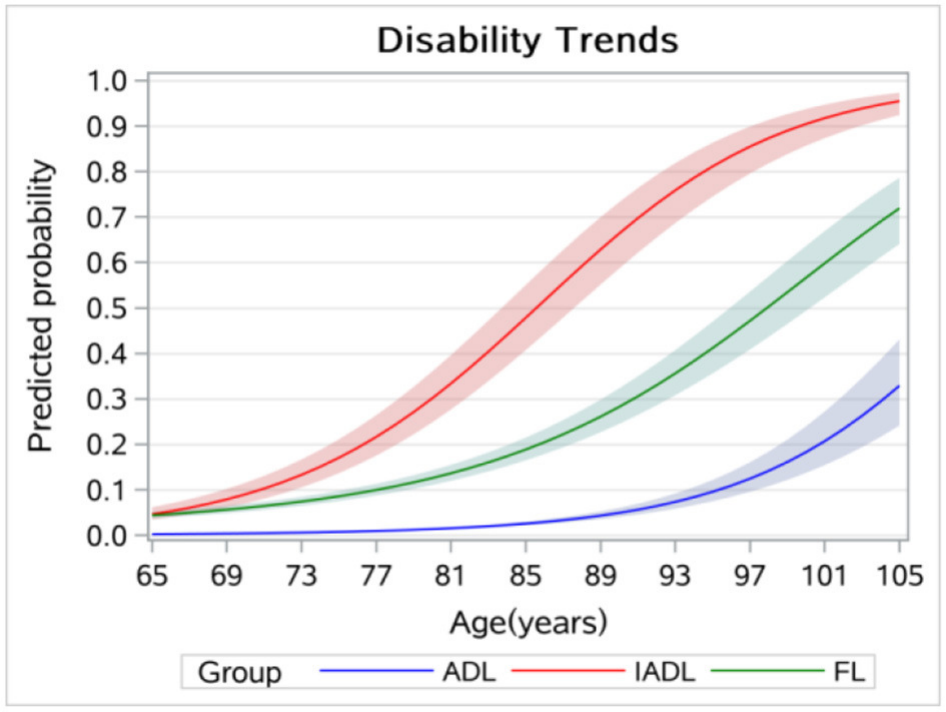
Age trends in disabilities among older adults.

Figure 1 displays the age trends of ADL, FL, and IADL disabilities, which increased from 0.003, 0.046, and 0.044 at the age of 65 years to 0.329, 0.719, and 0.955 at the age of 105 years, respectively. Overall, the age trend for IADL disability was the highest among the three kinds of disabilities, followed by the FL disability, and the age trend for ADL disability was the lowest.

**Figure 2:**
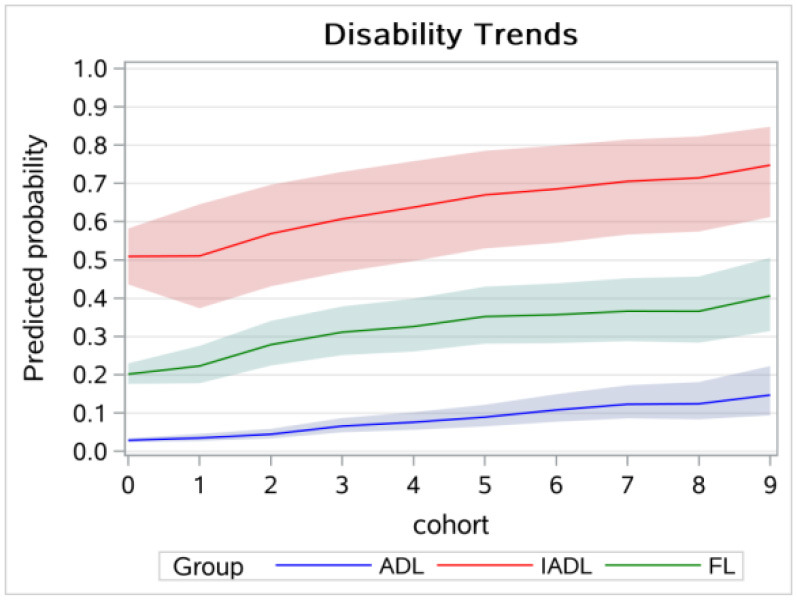
Cohort trends in disabilities among older adults.

Figure 2 displays the cohort trends of ADL, FL, and IADL disabilities, with 0 to 9 representing the 1905 and earlier cohort and the 1946 and subsequent years cohort, respectively. The results showed that the cohort trends of ADL, FL, and IADL disabilities increased from 0.029, 0.201, and 0.509 in the 1905 and earlier cohort to 0.147, 0.406, and 0.748 in the 1946 and above cohort, respectively. Overall, the cohort trend of IADL disability was the highest among the three types of disabilities, followed by FL disability, and ADL disability had the lowest cohort trend.

Table 2 shows the contributions of disablement process factors to cohort trends in ADL, IADL, and FL disabilities. As for risk factors, gender had made a considerable contribution to disability and negatively contributed to cohort trends in FL and IADL disabilities, and the contributions decreased from −14.7 and −347.1% in the 1906–2010 cohort to −9.9% and −21.7% in the 1946 and above cohort. Except for the 1906–2010 cohort, residence also made sizable contributions and positively contributed to the cohort trends in ADL disability among older adults, and the contributions increased from 3.3% in the 1911–1915 cohort to 17.8% in 1946 and later cohort. Education was also an important contributor and negatively contributed to the cohort trends in disability, and overall, the contributions were larger in more recent cohorts. Health behaviors also significantly contributed to cohort trends in disability. Smoking positively contributed to the disability trend, and the contributions to FL and ADL increased from 5.6 and 3.3% in the 1906–2010 cohort to 9.8 and 7.4% in the 1946 and above cohort, and the contributions (except for the 1906–2010 cohort) to IADL increased from 5.1% in the 1911–1915 cohort to 14.5% in the 1946 and above cohort. Exercise positively contributed to the cohort trends in ADL and IADL disabilities, and the effects of IADL disability decreased from 135.3 to 11.4%. Exercise also negatively contributed to the cohort trend in FL disabilities, and the contributions increased from −1.4% in the 1906–2010 cohort to −24.5% in the 1946 and after cohort. Overall, chronic disease positively contributed to the increment of cohort trends in disability, and the contribution had increased from 0.7, −0.7, and 0.0% in the 1906–2010 cohort to 30.0, 26.0, and 38.5% in the 1946 and later cohort. Psychological resilience had relatively few contributions to cohort trends in disability. As for accommodating factors, family income made a positive contribution to the increment of cohort trends in ADL disability but a negative contribution to cohort trends in FL and IADL disabilities. Having a spouse showed negative effects on ADL disability in the earlier cohorts, which then turned into a positive one, from 52.9 to −12.3% in later cohorts. Living arrangements and SP made relatively minor contributions to cohort effects in disability.

**Table 2:** The contributions of disablement process factors to ADL, IADL, and FL disabilities trends.

**Cohorts**	**Risk factors**				**Disablement process**			**Accommodating factors**			
	**Gender**	**Residence**	**Education**	**Smoking**	**exercise**	**Chronic disease**	**Psychological resilience**	**Living arrangement**	**Having a spouse**	**Family income**	**SP**
**FL (ref: 1905 and before)**											
1906–2010	−0.147	0.007	−0.014	0.056	−0.022	0.007	0.147	−0.007	0.028	−0.308	0.035
1911–1915	−0.134	−0.002	−0.059	0.006	−0.066	−0.002	0.008	−0.016	0.022	−0.198	0.043
1916–1920	−0.116	−0.007	−0.062	0.004	−0.088	0.046	−0.033	−0.012	0.012	−0.228	0.034
1921–1925	−0.126	−0.005	−0.060	0.019	−0.129	0.104	−0.019	−0.016	−0.012	−0.265	0.044
1926–1930	−0.105	−0.009	−0.082	0.031	−0.134	0.163	−0.042	−0.021	−0.026	−0.292	0.031
1931–1935	−0.085	−0.011	−0.093	0.050	−0.114	0.216	−0.070	−0.024	−0.040	−0.341	0.028
1936–1940	−0.090	−0.015	−0.153	0.066	−0.108	0.261	−0.082	−0.022	−0.054	−0.387	0.012
1941–1945	−0.104	−0.016	−0.230	0.077	−0.134	0.295	−0.101	−0.021	−0.078	−0.458	−0.016
1946 and after	−0.099	−0.018	−0.245	0.098	−0.159	0.300	−0.076	−0.018	−0.070	−0.473	−0.048
**ADL (ref: 1905 and before)**											
1906–2010	−0.023	−0.069	0.000	0.033	0.092	−0.007	0.056	−0.003	0.003	0.246	0.013
1911–1915	−0.042	0.033	−0.005	0.009	0.119	−0.005	0.011	−0.057	0.004	0.225	0.026
1916–1920	−0.034	0.068	−0.006	0.006	0.098	0.028	−0.012	−0.033	0.003	0.211	0.020
1921–1925	−0.042	0.057	−0.006	0.016	0.114	0.090	−0.002	−0.055	−0.005	0.240	0.028
1926–1930	−0.035	0.085	−0.008	0.028	0.099	0.154	−0.013	−0.068	−0.010	0.268	0.021
1931–1935	−0.024	0.116	−0.009	0.043	0.087	0.191	−0.029	−0.069	−0.014	0.291	0.021
1936–1940	−0.028	0.139	−0.016	0.051	0.083	0.230	−0.033	−0.066	−0.018	0.319	0.005
1941–1945	−0.033	0.154	−0.024	0.056	0.101	0.248	−0.047	−0.061	−0.024	0.354	−0.014
1946 and after	−0.040	0.178	−0.028	0.074	0.114	0.260	−0.041	−0.060	−0.021	0.382	−0.028
**IADL (ref: 1905 and before)**											
1906–2010	−3.471	0.000	−0.706	0.647	1.353	0.000	1.471	−0.235	0.529	−3.176	0.588
1911–1915	−0.674	−0.005	−0.274	0.051	0.312	−0.014	0.093	−0.065	0.107	−0.563	0.228
1916–1920	−0.421	−0.007	−0.201	0.026	0.208	0.087	0.019	−0.035	0.052	−0.492	0.130
1921–1925	−0.342	−0.006	−0.153	0.050	0.206	0.156	0.038	−0.035	−0.012	−0.474	0.131
1926–1930	−0.276	−0.007	−0.191	0.061	0.161	0.229	−0.002	−0.041	−0.044	−0.493	0.098
1931–1935	−0.227	−0.009	−0.204	0.088	0.129	0.281	−0.033	−0.040	−0.060	−0.543	0.094
1936–1940	−0.220	−0.010	−0.311	0.107	0.108	0.334	−0.039	−0.036	−0.088	−0.597	0.055
1941–1945	−0.234	−0.012	−0.433	0.118	0.131	0.372	−0.061	−0.034	−0.128	−0.687	0.020
1946 and after	−0.217	−0.013	−0.469	0.145	0.114	0.385	−0.044	−0.031	−0.123	−0.721	−0.040

### Sensitivity analysis

**Figure 3:**
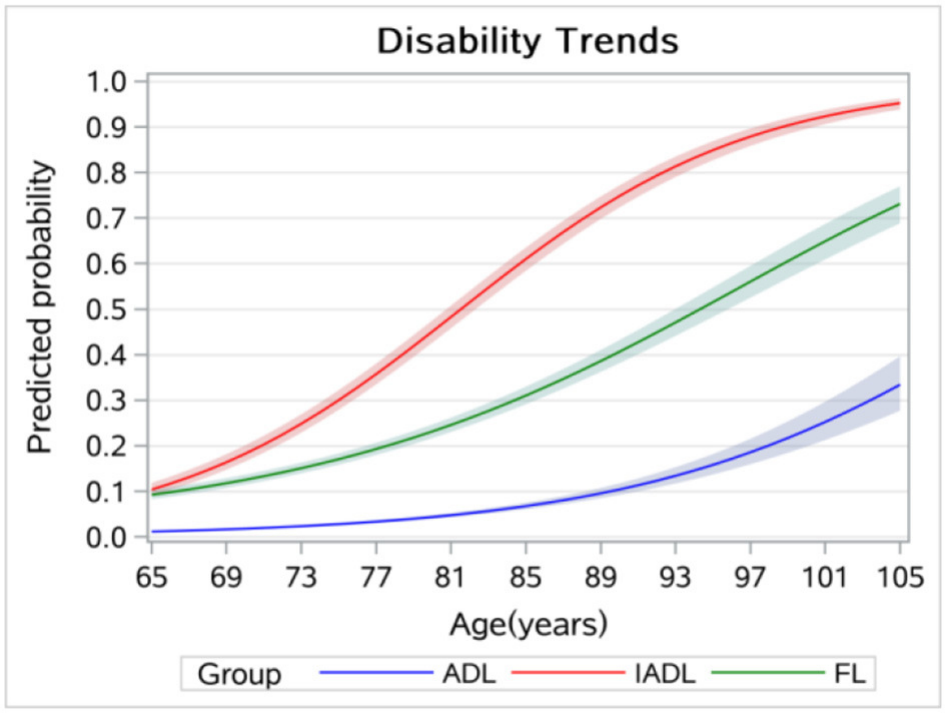
Age trends in disability without controlling for cohort effects.

**Figure 4:**
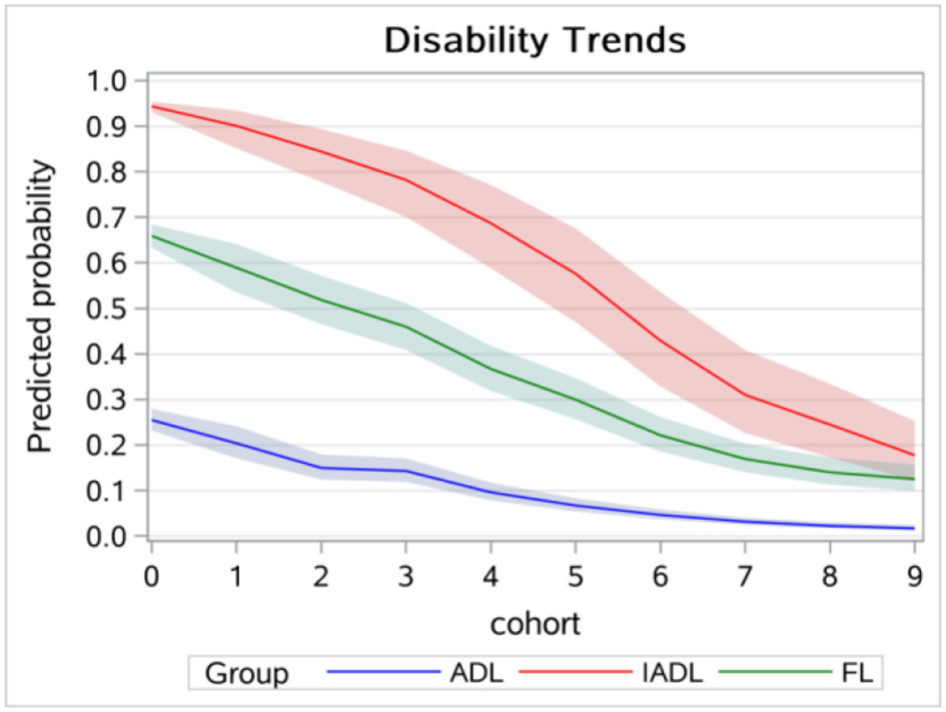
Cohort trends in disability without controlling for age effects.

Figure 3 displays the age trends in disability without controlling for the cohort effect, which showed a higher disability level at the age of 65 years and a lower increasing rate compared to Figure 1. Figure 4 displays the results of the cohort trends in disability without controlling for the age effect and the results showed declining cohort trends, while the results in Figure 2 showed increasing cohort trends.

## Discussions

This study explored age and cohort trends in disability and related factors among Chinese older adults. We found increasing age and cohort trends in ADL, IADL, and FL disabilities, and the results of the sensitivity analysis indicated that neglecting cohort or age effect can cause significant bias when estimating time trends in disability. More specifically, the age trend in FL was lower than IADL but higher than ADL. We also found that IADL disability and FL began earlier than ADL disability. The results were consistent with those of other studies (19, 23). According to the disablement process model, FL eventually leads to ADL and IADL disabilities (10). In this study, the results further indicated that FL was more likely to result in IADL disability than ADL disability. Our results have policy implications, and health policies, such as the Health Country Action in China and other countries similar to China, should pay more attention to identifying and reducing FL to prevent disabilities, especially IADL disability.

Older adults in more recent cohorts suffered more from disabilities after controlling for age and other disablement process factors. The results were inconsistent with those of Zhang's studies conducted in China, which found decreasing cohort trends in disabilities (8). Two reasons may explain the inconsistent results: one was because a different method was used to estimate the A-P-C model. Zhang's study used an IE estimator, while the current study used the hierarchical logistic model. The other difference between the two may be that Zhang's study focused on the oldest-old, while our study focused on older adults younger than those in Zhang's study. According to our study, health policies such as long-term health insurance should increase the financial support for older adults in a younger cohort. In our study, we found decreasing trends in disabilities in descriptive analysis and sensitivity analysis, and the main reason was that the age effect was not controlled for and younger age was more likely to be part of the earlier cohorts.

Based on the disablement process model, we further explored the contributors of cohort trends in disability. As for demographic factors, gender negatively contributed to cohort trends in IADL disability and FL, which was consistent with what was observed in another study (15). We also found that the contributions decreased in more recent cohorts. The improvement in the social-economic status of women in more recent cohorts may explain the result. Hence, health services should pay more attention to the health demands of older women in earlier cohorts. Residence positively contributed to the cohort trends in ADL disability among older adults, and the contributions were larger in more recent cohorts. The development of urbanization has expanded the gap between urban and rural public resources. This calls for a more equitable distribution of health resources between rural and urban areas. Education reduced the increasing trends in disability, which was consistent with observations from another study (24). Moreover, we also found that the negative contributions were larger in more recent cohorts. This may be because older adults in more recent cohorts obtained more education and had more health resources or health knowledge to maintain functional health in later life. Therefore, more health services should be provided for older adults in earlier cohorts.

As for health behaviors, drinking positively contributed to the cohort trends in disability with larger contributions in more recent cohorts. Exercise negatively contributed to the cohort trends in FL disability, and the effects were larger in more recent cohorts. The results were inconsistent with those of Chen and Frank's study, which only found minor contributions of health behavior to disability trends (15). The difference might have been caused by Chen and Frank only using two-time point data. Older adults were more likely to smoke and exercise in more recent cohorts, which may explain these results. However, exercise also positively contributed to cohort trends in ADL and IADL disabilities, and the contributions increased in more recent cohorts. This outcome could be because disabled older adults were more likely to change their unhealthy lifestyles. Therefore, health policies, such as the Health Country Action in China and other countries similar to China, should promote exercise in older adults in earlier cohorts and reduce smoking in more recent cohorts.

As for social support, family income made the largest contribution to cohort trends in IADL and ADL disabilities, showing a negative contribution to cohort trends in IADL disability, but a positive contribution to the increment of cohort trends in ADL disability. The results were inconsistent with those of Zajacova's studies, which only found declining trends in economic status, which were associated with increasing disability prevalence (24). The reason may be that Zajacova's study did not distinguish between ADL and IADL disabilities. Current results might be explained by the fact that higher family income was more likely to prevent mild disability (FL or IADL disability), while it can only maintain the lives of older adults with severe disability (ADL disability). The results indicated that families should provide timely support to prevent mild disability in older adults. Overall, the contributions of family income were larger in more recent cohorts. The results could be explained by the rising family income in more recent cohorts. As older adults had low family incomes, health services, such as integrated medical and nursing services, should be provided more affordably to older adults, especially in earlier cohorts. It is also necessary to promote the establishment of long-term care insurance in China and other countries similar to China to provide support for less severe disabilities and eventually improve the functional health of older adults and reduce the long-term care burden to the family. We only found minor contributions of living arrangement to cohort trends in disabilities and increasing negative contribution of having a spouse, which may be caused by fewer children in the family and the increase of “empty-nest” older adults in more recent cohorts. Thus, policies in China and other countries similar to China should encourage children to live with their parents in the future (e.g., providing allowances or tax benefits to children living with their parents).

For the disablement process, we found that chronic disease positively contributed to the escalations of cohort trends in disabilities. Previous studies showed that chronic disease had a large effect on disability trends among older adults (18, 25), which was consistent with the results of our study. Increases in chronic disease in more recent cohorts may explain these results. According to our studies, health policies such as the Health Country Action in China and other countries similar to China should conduct interventions for chronic disease in more recent cohorts to prevent disability among older adults.

## Limitations and strengths

This study has several limitations. First, as longitudinal data were used in our study, the period effect was not included in the A–P–C analysis. However, as our study focused on older adults, the span of the period was much shorter than age and cohort, so the effect of period could be trivial and omitted from the model (7). Second, although we used longitudinal data, we were still unable to isolate the causal direction of related factors of cohort trends in disability, such as exercise and family income. Third, the measurement of disability was gathered from self-reported surveys, which may cause bias. However, self-reported data are commonly used in disability research of older adults, which can reflect more accurately personal status interacting with the real world (26). Finally, our study only calculated the total contributions of each disablement process factor, which did not explore the relationships between these contributions. Therefore, more studies are needed to explore the mechanism of the contributions.

Nonetheless, this study contributes to the existing literature in four aspects. First, this study used five-wave national representative longitudinal data in a developing country, which allowed us to better investigate age and cohort trends in disability and explore contributors to cohort trends in disability. Second, it was conducted in the Chinese context, which fills the gap of lacking A–P–C analysis beyond the developed countries. Third, this study combined ADL, IADL, and FL to study age and cohort trends in disability and related contributors of cohort trends in disability, which can better reveal the disability trends and related contributors of disabilities. Finally, we also added the disablement process model by revealing the age and cohort trends in disability and the contributions of disablement process factors to cohort trends in disability.

## Conclusions

Our study draws the following conclusions. First, in light of the declining fertility rate and the rapidly aging society, our study showed that the likelihood of disability has increased in both age and cohort trends. This requires society to pay more attention to reducing disability among older adults. Second, our study gauged the relative importance of the disablement process factors in explaining cohort trends in disability, necessitating more precise interventions to reduce disability among older adults. Finally, our study divided temporal trends into two dimensions, age and cohort, and explored various effects of relevant factors on cohort trends in disability. It may invoke further research in this area across different cultural contexts and populations and provide interventions for disability among older adults of other countries similar to China.

## Data availability statement

The datasets presented in this study can be found in online repositories. The names of the repository/repositories and accession number(s) can be found in the article/supplementary material.

## Author contributions

CP: conceived, designed, analyzed the data, was responsible for the interpretation of findings, primary drafting of the manuscript, and revisions. NC, SL, and MK: made substantial contributions to the interpretation of data and revising the manuscript critically for important intellectual content. All authors read and approved the final manuscript.
